# Comparison of healing effectiveness of different debridement approaches for diabetic foot ulcers: a network meta-analysis of randomized controlled trials

**DOI:** 10.3389/fpubh.2023.1271706

**Published:** 2023-12-11

**Authors:** Peng Ning, Yupu Liu, Jun Kang, Hongyi Cao, Jiaxing Zhang

**Affiliations:** Department of Endocrine and Metabolism, Chengdu Fifth People’s Hospital (The Second Clinical Medical College, Affiliated Fifth People’s Hospital of Chengdu University of Traditional Chinese Medicine), Geriatric Diseases Institute of Chengdu, Chengdu, China

**Keywords:** debridement, diabetic foot ulcers, healing rate, area reduction, network meta-analysis, randomized controlled trial

## Abstract

**Objectives:**

The choice of the debridement method is very important for the healing of diabetic foot ulcers (DFUs), but the relative effectiveness of different debridement methods in the healing of DFUs remains unclear. This study conducted a network meta-analysis of the relative healing effectiveness of different debridement methods in patients with DFUs.

**Methods:**

We performed a literature search in PubMed, Embase, and Cochrane Library from database inception up to 30 June 2023 for screening randomized controlled trials on the healing effectiveness of debridement in DFUs. Outcome measures included ulcer healing rate and ulcer area reduction rate. The Cochrane Risk Bias Tool, version 2.0, was used to assess the risk of bias in the included trials. R software was used for performing statistical analysis and GraphPad Prism was used for image plotting.

**Results:**

A total of 19 randomized controlled trials were included, and 900 patients with DFUs were assessed in this analysis. The proteolytic fraction from the latex of *Vasconcellea cundinamarcensis* (P1G10) in enzymatic debridement showed the best ulcer healing rate (SURCA = 0.919) when compared with the standard of care (SOC) group, with a mean difference (MD) and 95% confidence interval (CI) of 1.40 (0.57, 2.36). Kiwifruit extract demonstrated the best effect on the ulcer area reduction rate (SURCA = 0.931), when compared with that in the SOC group, with an MD and 95% CI of 0.47 (0.27, 0.66).

**Conclusion:**

Enzymatic debridement was superior to other debridement methods in terms of ulcer healing rate and ulcer area reduction rate in patients with DFUs. However, as the quality of the included trials is low, enzymatic debridement can be used as a candidate debridement method in addition to sharp-based debridement in clinical practice.

**Systematic review registration:**

https://www.crd.york.ac.uk/prospero/display_record.php?ID=CRD42023441715.

## 1 Introduction

The prevalence of diabetic foot ulcers (DFUs) has steadily increased. The International Diabetes Foundation estimated that 40–60 million people worldwide have DFUs (1). If left untreated, DFUs can progress to soft tissue infections and gangrene, resulting in limb loss (2). The latest meta-analysis revealed that DFUs are associated with a high overall mortality rate of nearly 50% within 5 years (3), posing a grave threat to patients’ wellbeing. DFUs typically arise from a combination of factors, including prolonged hyperglycemia, neuropathy, and vascular disease (4). DFU management involves various aspects, such as wound debridement, infection control, and ulcer healing. Wound debridement is considered a crucial intervention in DFU management, as it accelerates ulcer healing and reduces the risk of severe complications. The process involves eliminating non-viable wound bed and wound edge tissue, including excess callus, non-viable dermal tissue, foreign substances, and bacterial components, to promote wound healing (5). Currently, several approaches for debridement are available, such as mechanical debridement, including sharp debridement, surgery, wet-to-dry (6), ultrasound (7), hydrosurgery (8), or biological debridement (maggot debridement therapy) (9), and non-mechanical debridement, including autolytic (hydrogel (10) or alginate (11)) or biochemistry debridement (enzymatic) (12). While experts universally recognize the significance of regular wound debridement for enhancing DFU healing, available evidence supporting the most effective debridement method remains limited.

In May 2023, the International Working Group on the Diabetic Foot (IWGDF) (13) significantly updated the guidelines for DFU diagnosis and treatment. The guidelines emphasize that no debridement method can fully replace sharp instrument debridement, which remains the gold standard approach. Early aggressive initial and sequential debridement is essential for ulcer care. However, the specific approach to debridement may vary based on individual patient and ulcer characteristics, as well as cost and convenience considerations (14). While sharp debridement is highly recommended, the guidelines do not clearly recommend the relative effectiveness of other debridement methods for patients with DFUs. Therefore, to address this knowledge gap, a network meta-analysis (NMA) that integrates and assesses existing research data from randomized controlled trials (RCTs) was conducted to evaluate the relative healing effectiveness of different debridement methods in patients with DFUs. This NMA aimed to provide more specific guidance for clinical practice, offering a more reliable foundation for future research and treatment strategies. Thus, the objective of this study was to improve the treatment outcomes for patients with DFUs, reduce their suffering, and minimize the risk of complications.

## 2 Methods

### 2.1 Registration

This NMA adhered to the Preferred Reporting Items for Systematic Reviews and Meta-Analyzes (PRISMA) statement for systematic evaluation and meta-analysis (15). The study protocol was registered with the International Prospective Systems Evaluation Register (PROSPERO; registration no. CRD42023441715).

### 2.2 Search strategies

A comprehensive search was performed in three electronic databases (PubMed, EMBASE, and Cochrane Library) using a search strategy centered around the PICOS tool: Population (patients with DFU), Intervention (mechanical or non-mechanical debridement), and type of study (RCT). Supplementary Table S1 in the Additional Materials section presents the full list of search terms. Additionally, the reference lists of previous systematic reviews and meta-analyzes in this field were checked to identify any missing articles. All retrieved documents were stored in the EndNote version X9 database (Thomson ResearchSoft, Stanford, CA, United States).

### 2.3 Research selection

Studies that met the following inclusion criteria were selected (1): Subjects: adult patients with a confirmed diagnosis of type 1 or type 2 diabetes, wherein the diagnosis was made based on the World Health Organization 1999 and American Diabetes Association standards (16), meeting the IWGDF 2023 standard diagnosis for patients with DFU (17). No restrictions were imposed on nationality or race, and patients with gestational diabetes mellitus were not included in the test process. (2) Intervention measures: The experimental group underwent debridement using mechanical or non-mechanical debridement methods with no limitation on the treatment course and debridement frequency; (3) Control measures: standard of care (SOC); (4) Research type: RCT.

Studies that met the following exclusion criteria were not selected: (1) review articles, systematic evaluation, abstracts, conference papers, retrospective studies, cross-sectional studies, and cross-RCTs; (2) studies lacking relevant outcome indicators or extractable data; (3) animal and cell studies; (4) studies on patients with gestational diabetes; and (5) non-English literature.

Two researchers (PN and YL) independently screened the titles and abstracts to identify potentially eligible articles. Subsequently, a full-text search was performed to include eligible articles. In case of conflicting opinions, a third researcher (HC) made the final decision on selecting conflicting articles.

### 2.4 Data extraction

Data were extracted using a predesigned spreadsheet. Two researchers (PN and YL) independently extracted data from the included studies, including information on author, year, country, sample size, comparison, treatment details (various types of debridement and SOC), and outcome indicators (ulcer healing rate and ulcer area reduction rate). When outcome indicators were presented only in images, GetData Graph Digitizer 2.25 (GetData Software Development Company, Sydney, Australia) was used to collect the data of outcome indicators. Any discrepancies in the extracted data were resolved by a third researcher (JZ).

### 2.5 Risk-of-bias assessment

The Cochrane Risk Bias Tool, version 2.0, was used to assess the risk of bias in the included studies. This involved evaluating random sequence generation (selection bias), allocation concealment (selection bias), blinding of participants and personnel (performance bias), blinding of outcome assessment (detection bias), incomplete outcome data (attrition bias), selective reporting (reporting bias), and other bias. Study quality was categorized as low, unclear, or high risk of bias. Two researchers (YL and JK) independently evaluated all studies, and disagreements between the two researchers were resolved by a third researcher (JZ).

### 2.6 Data analysis

Statistical analysis was conducted using R software, version 4.3.1 (R Core Team, Vienna, Austria) and the packages “netmeta,” “gemtc,” and “rjags” (18) were used; the image plotting was performed using GraphPad Prism, version 9.4.1 (GraphPad Software, San Diego, CA, United States). First, we constructed a network plot as a simple overview to display all available evidence for each intervention. Second, we performed Bayesian network analysis based on Markov Chain Monte Carlo (18) to analyze the ulcer healing rate and ulcer area reduction rate in patients with DFUs for different debridement approaches. The number of tuning and simulation iterations was set at 5,000 and 20,000, respectively. An I^2^ value of ≤50% indicated minimal or no heterogeneity between studies, leading to the adoption of the fixed-effects model. Otherwise, the random-effects model was used (19). The results of the generated League Table were expressed using mean difference (MD) and 95% confidence interval (CI), and the data were not statistically significant when the 95% CI value contain 0. Then, to assess the probability of each intervention being the most effective, the surface under the cumulative ranking curve (SUCRA) was calculated (with a value ranging from 0 to 1). A higher SUCRA value indicated a greater likelihood of a treatment being highly effective or having the highest level of effectiveness, thereby maximizing the potential for achieving optimal outcome indicators (20). Finally, heterogeneity analysis was conducted for all included studies, wherein heterogeneity was considered to exist when I^2^ > 50%. A value of *α* = 0. 05 was considered to indicate statistical significance.

## 3 Results

### 3.1 Research selection and characteristics

**Figure 1:**
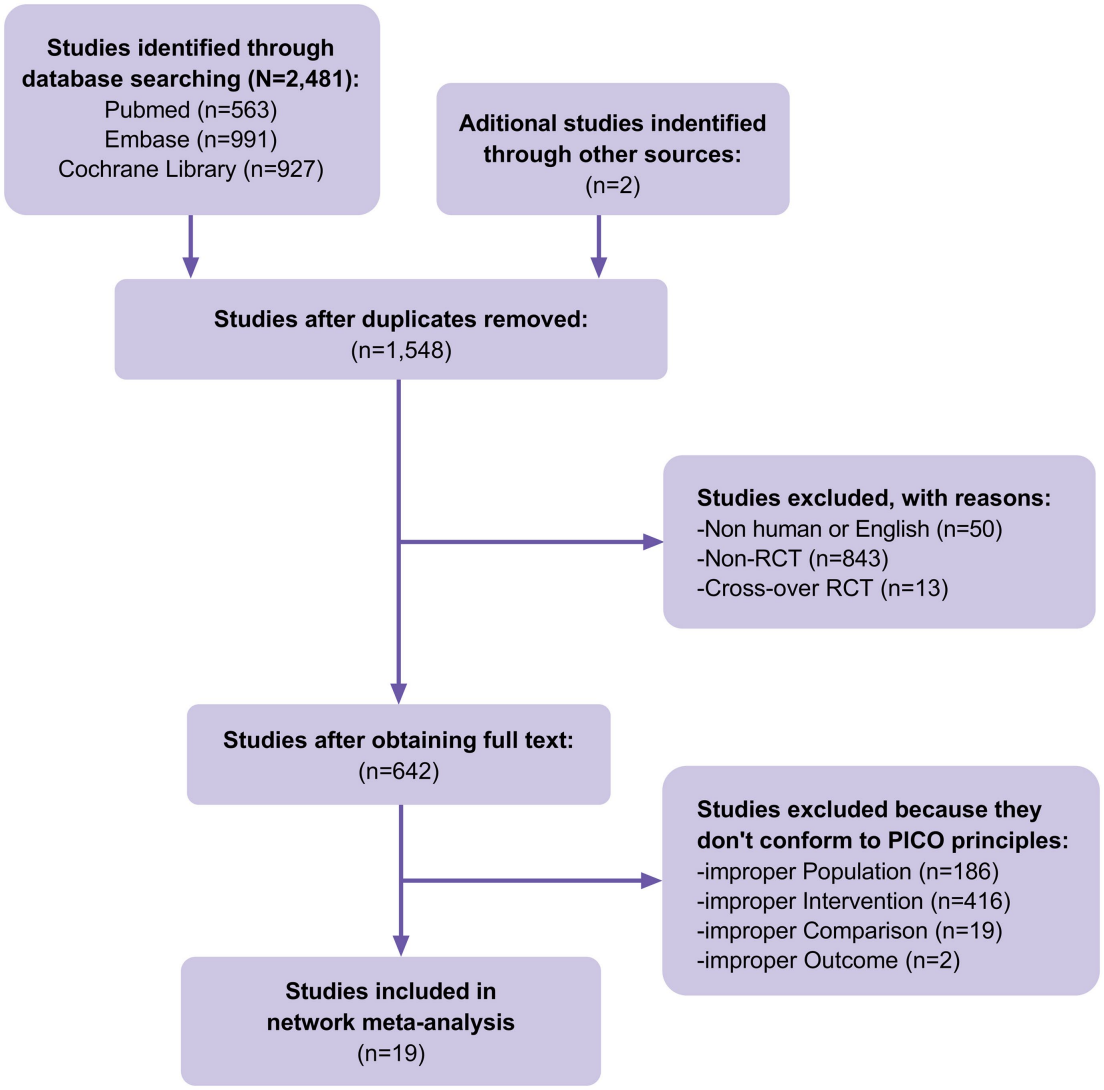
Flow of trials through the review.

A total of 2,481 studies were identified in our initial database search, covering the period from the inception of the databases to 30 June 2023. Additionally, when manually searching the reference lists of previous systematic reviews and meta-analyzes, two additional studies that met the inclusion criteria were identified. A total of 1,548 studies were excluded after eliminating duplicate articles. Fifty studies were excluded because they were non-human or non-English studies, 856 were excluded because they were non-RCTs or cross-RCTs, and 623 were excluded because they did not conform to the PICO principles of the study. Finally, 19 RCTs (Figure 1) involving 900 patients with DFUs were included in the analysis. The baseline characteristics of the population are listed in Table 1. The advantages and disadvantages of different debridement methods are shown in Table 2. The debridement methods reported in the selected studies mainly include sharp debridement, surgical debridement, ultrasonic debridement, enzymatic debridement, and autolytic debridement.

**Table 1:** Characteristics of RCTs on the effectiveness of debridement in patients with diabetic foot ulcers.

Study	Region	Criteria	Type	Intervention			Comparison			Follow-up	Outcome
				*N*	Ulcer area(cm^2^)	Method	*N*	Ulcer area(cm^2^)	Method		
Piaggesi et al. (21)	Italy	Wagner	Neuropathic	21	NA	Surgical + SOC	20	NA	Sharp + SOC	6 months	H
Ennis et al. (22)	USA	Wagner	NA	27	NA	Ultrasonic + SOC	28	NA	SOC	3 months	H
Amini et al. (23)	Iran	Wagner	Neuropathic or Neuroischemic	20	6.8 ± 3.5	Ultrasonic + SOC	20	9.9 ± 7.6	SOC	6 months	H, A
Yao et al. (24)	USA	Wagner	Neuropathic	8	2.2 ± 1.7	Ultrasonic + SOC	4	2.1 ± 0.9	SOC	5 weeks	A
Michailidis et al. (25)	Australia	UTWCS	NA	5	NA	Ultrasonic + SOC	5	NA	Sharp + SOC	6 months	H
Bajpai et al. (26)	USA	NA	NA	4	NA	Ultrasonic + SOC	4	NA	SOC	3 months	H, A
Lázaro-Martínez et al. (27)	Spain	UTWCS	Neuropathic	27	7.5 ± 7.6	Ultrasonic + SOC	24	4.2 ± 3.3	Surgical + SOC	6 months	H, A
Tallis et al. (28)	USA	BWAT	Neuropathic	24	3.0 ± 2.1	COO + Sharp	24	2.4 ± 2.1	Sharp	3 months	H, A
Galperin et al. (29)	USA	Wagner	NA	9	8.1 ± 8.1	CCO	8	7.8 ± 6.9	Hydrogel	1 month	A
Motley et al. (30)	USA	UTWCS	Neuropathic	28	2.0 ± 1.1	COO + Sharp	27	1.8 ± 1.6	Hydrogel + Sharp	3 months	H, A
Lantis et al. (31)	USA	Wagner	Neuropathic	88	3.0 ± 3.6	Trial 1: COO	86	2.5 ± 3.2	Trial 1: SOC	3 months	A
						Trial 2: COO			Trial 2: Hydrogel		
						Trial 3: COO			Trial 3: Sharp		
						Trial 4: COO + Sharp			Trial 4: Sharp		
Tonaco et al. (32)	Brazil	NA	Neuropathic	27	NA	P1G10	23	NA	Hydrogel	4 months	H
Mohajeri et al. (33)	Iran	NA	Neuropathic	17	4.2 ± 0.9	Kiwifruit extract + Sharp	20	4.0 ± 0.7	SOC + Sharp	3 weeks	A
Kardoust et al. (34)	Iran	Wagner	Neuropathic	9	2.2 ± 0.7	Kiwifruit extract	9	2.0 ± 0.6	SOC	1 month	A
Jensen et al. (35)	USA	Wagner	NA	14	NA	Hydrogel + Sharp	17	NA	SOC + Sharp	5 months	H
Djavid et al. (36)	Iran	Wagner	Neuropathic	30	3.1 ± 2.5	Hydrogel	31	3.5 ± 4.2	SOC	6 months	H, A
Della Pepa et al. (37)	Italy	UTWCS	Neuropathic, Ischemic, or Neuroischemic	20	2.1 ± 1.8	Hydrogel + Sharp	20	2.3 ± 2.7	SOC + Sharp	3 months	H, A
Donaghue et al. (38)	USA	Wagner	NA	50	2.6 ± 0.5	Alginate	25	3.0 ± 0.6	SOC	2 months	H, A
Lalau et al. (39)	France	NA	Neuropathic	39	8.0 ± 10.5	Alginate	38	8.8 ± 16.0	SOC	6 weeks	H, A

UTWCS, University of Texas Wound Classification System; BWAT, Bates-Jensen Wound Assessment Tool; COO, Clostridial collagenase ointment; SOC, Standard of care; P1G10, Proteolytic Fraction from Latex of *Vasconcellea cundinamarcensis*; H, healing rate; A, area reduction rate.

**Table 2:** Advantages and disadvantages of different debridement methods.

Method	Explanation	Advantages	Disadvantages
Mechanical debridement			
Sharp debridement	Use a sharp tool, such as a scalpel or scissors, to remove dead tissue in the dressing room	Guideline recommendation Quick Specific Less costly	Painful Large injury
Surgical debridement	Use surgical techniques to remove necrotic tissue in the operating room	Clear thoroughly Deep-tissue samples were collected for pathological examination. Provide a clean bed for future operations such as transplants or flaps	High technical requirements Painful Larger injury Expensive
Wet-to-dry debridement (6)	Gauze moistened with salt water is allowed to dry and removed with dead tissue	Quick	Non-specific Easily damages healthy tissue Painful
Ultrasound debridement (7)	Use the sound energy generated by ultrasound to remove dead tissue	Quick Specific	May cause pain Expensive
Hydrosurgery (8)	The wound is irrigated with high-pressure water, either manually or with a mechanical spray device	Quick Suited for larger wounds	Non-specific Cross-infection May cause pain Expensive
Biological debridement (maggot debridement therapy) (9)	The sterile maggots were placed directly on the infected area and wrapped with close net dressing while actively avoiding healthy tissue	Relatively quick Ultraspecific	Difficulty accessing maggots May cause minor pain Patients may resist for psychological reasons
Non-mechanical			
Autolytic debridement (hydrogel (10) or alginate (11))	Relies on a dressing type that permits the wound to remain moist and facilitates autolysis of the devitalized tissue	Convenient and simple Selective for non-viable tissue Minimal or no discomfort Less costly	Slow process May lead to softening of the surrounding tissue and infection Not ideal for heavily infected wounds
Biochemistry (enzymatic debridement) (12)	Uses the application of enzymes such as collagenase to help lyse non-viable tissue	Convenient and simple Selective and specific for non-viable tissue Minimal or no discomfort	Slow process May cause allergic reactions or other discomfort Not ideal for heavily infected wounds

### 3.2 Risk of bias in studies

**Figure 2:**
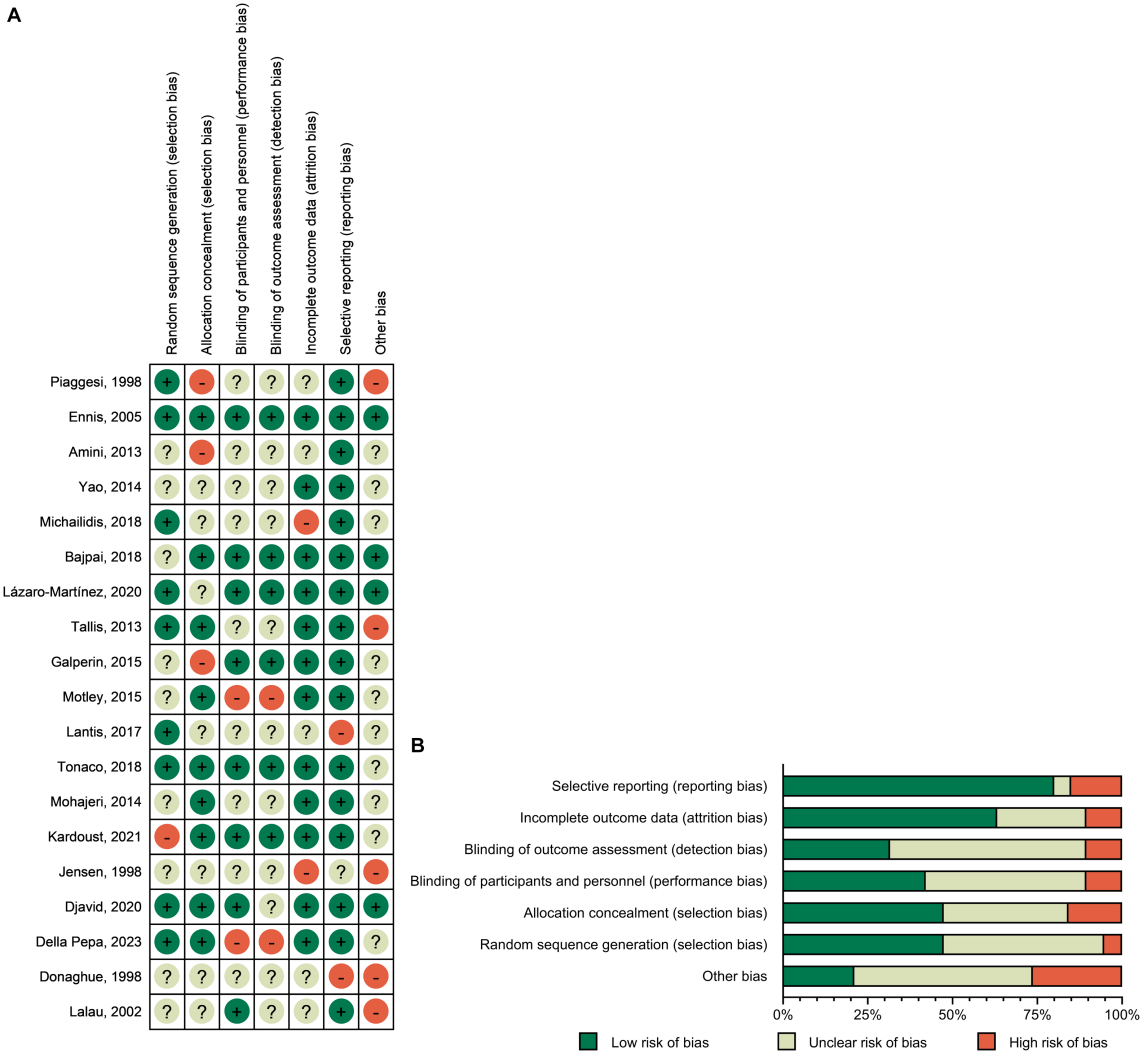
Risk-of-bias graph: **(A)** Risk-of-bias summary: review authors’ judgments about each risk-of-bias item for each included study. **(B)** Risk-of-bias graph: judgments about each risk-of-bias item presented as percentages across all included studies.

The risk of bias was assessed for all the included RCTs. Among the 19 studies, 10 did not specifically describe the generation of random sequences, 10 did not specifically describe the concealment scheme of RCTs, 11 did not specifically describe the method of blinding participants and implementers, and 13 did not specifically describe the blinding scheme of the outcome measure. The data reported in 12 studies were incomplete, those in 3 studies might have been selectively reported, and those in 15 studies may have had reporting bias. The risk-of-bias plots showed individual and overall document-level quality separately (Figure 2).

### 3.3 Healing rate

**Figure 3:**
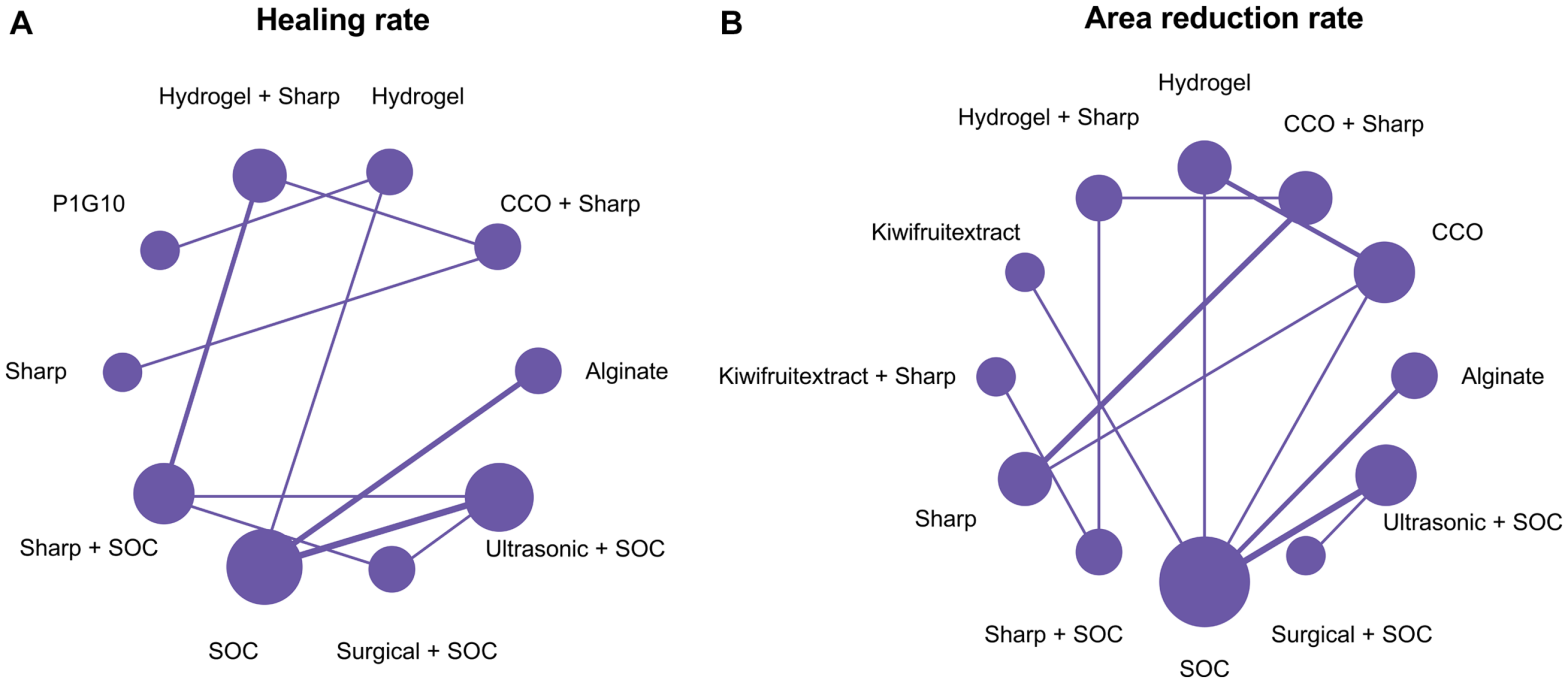
Network plots. **(A)** Healing rate; **(B)** Area reduction rate. COO, Clostridial collagenase ointment; SOC, Standard of care; P1G10, Proteolytic Fraction from Latex of *Vasconcellea cundinamarcensis*.

**Figure 4:**
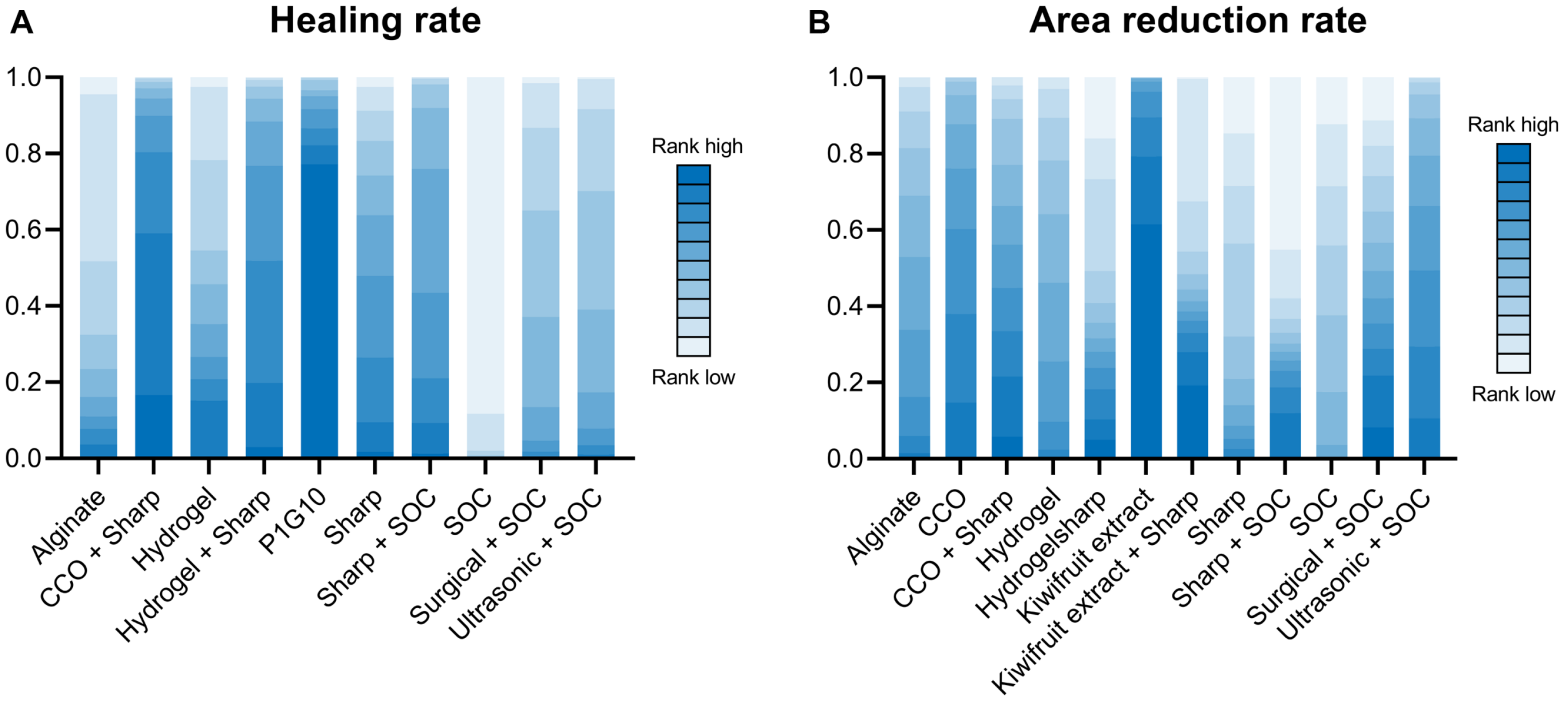
Cumulative probability ranking graph. **(A)** Healing rate; **(B)** Area reduction rate. COO, Clostridial collagenase ointment; SOC, Standard of care. P1G10, Proteolytic Fraction from Latex of *Vasconcellea cundinamarcensis*.

A total of 14 RCTs investigated the effect of different debridement procedures on the ulcer healing rate in patients with DFU. A network diagram of the ulcer healing rate is shown in Figure 3A. The heterogeneity test indicated an I^2^ value of 23%; hence, the fixed-effects model was used. According to SUCRA analysis and the League Table (Table 3A), the proteolytic fraction from the latex of *Vasconcellea cundinamarcensis* (P1G10) in enzymatic debridement showed the best effect on the ulcer healing rate in patients with DFUs (SUCRA = 0.919). The cumulative probability ranking graph is shown in Figure 4A, with an MD and 95% CI of 1.40 (0.57, 2.36) when compared with the SOC group.

**Table 3:** Mean difference (MD) and 95% confidence interval (CI) for outcome measures.

A. Healing rate									
Alginate									
−0.59 (−1.46, 0.25)	CCO + Sharp								
−0.17 (−0.92, 0.53)	0.41 (−0.51, 1.34)	Hydrogel							
−0.50 (−1.33, 0.30)	0.09 (−0.15, 0.36)	−0.32 (−1.21, 0.57)	Hydrogel + Sharp						
−1.05 (−2.09, −0.10)	−0.45 (−1.64, 0.66)	−0.86 (−1.64, −0.25)	−0.55 (−1.71, 0.54)	P1G10					
−0.39 (−1.30, 0.51)	0.20 (−0.07, 0.53)	−0.21 (−1.18, 0.76)	0.11 (−0.27, 0.51)	0.66 (−0.49, 1.89)	Sharp				
−0.37 (−1.12, 0.34)	0.22 (−0.23, 0.68)	−0.19 (−1.01, 0.62)	0.12 (−0.25, 0.51)	0.67 (−0.36, 1.78)	0.01 (−0.53, 0.55)	Sharp + SOC			
0.35 (−0.06, 0.82)	0.95 (0.24, 1.70)	0.53 (−0.01, 1.15)	0.85 (0.20, 1.57)	1.40 (0.57, 2.36)	0.75 (−0.03, 1.55)	0.73 (0.18, 1.34)	SOC		
−0.17 (−0.89, 0.52)	0.42 (−0.08, 0.94)	0.00 (−0.78, 0.81)	0.32 (−0.11, 0.78)	0.87 (−0.13, 1.97)	0.22 (−0.37, 0.81)	0.19 (−0.01, 0.46)	−0.53 (−1.10, −0.01)	Surgical + SOC	
−0.18 (−0.86, 0.47)	0.41 (−0.12, 0.96)	0.00 (−0.75, 0.77)	0.31 (−0.15, 0.80)	0.86 (−0.11, 1.93)	0.21 (−0.41, 0.82)	0.19 (−0.10, 0.50)	−0.54 (−1.07, −0.08)	−0.01 (−0.25, 0.23)	Ultrasonic + SOC

B. Area reduction rate											
Alginate											
−0.08 (−0.21, 0.04)	CCO										
−0.06 (−0.47, 0.35)	0.02 (−0.37, 0.41)	CCO + Sharp									
0.01 (−0.13, 0.16)	0.10 (0.01, 0.19)	0.08 (−0.33, 0.48)	Hydrogel								
0.18 (−0.66, 1.03)	0.27 (−0.57, 1.10)	0.25 (−0.50, 0.99)	0.17 (−0.68, 1.01)	Hydrogel + Sharp							
−0.33 (−0.55, −0.11)	−0.25 (−0.45, −0.04)	−0.27 (−0.71, 0.18)	−0.35 (−0.56, −0.13)	−0.52 (−1.38, 0.35)	Kiwifruit extract						
0.15 (−1.08, 1.36)	0.23 (−0.99, 1.44)	0.21 (−0.94, 1.36)	0.13 (−1.09, 1.35)	−0.04 (−0.91, 0.83)	0.48 (−0.76, 1.71)	Kiwifruit extract + Sharp					
0.17 (−0.23, 0.57)	0.25 (−0.13, 0.64)	0.23 (0.14, 0.33)	0.15 (−0.23, 0.55)	−0.02 (−0.76, 0.73)	0.50 (0.07, 0.94)	0.02 (−1.13, 1.18)	Sharp				
0.32 (−0.90, 1.53)	0.40 (−0.81, 1.61)	0.38 (−0.76, 1.52)	0.30 (−0.91, 1.51)	0.14 (−0.73, 1.00)	0.65 (−0.58, 1.88)	0.17 (0.03, 0.31)	0.15 (−0.99, 1.29)	Sharp + SOC			
0.13 (0.03, 0.24)	0.22 (0.15, 0.28)	0.20 (−0.20, 0.60)	0.12 (0.02, 0.22)	−0.05 (−0.89, 0.79)	0.47 (0.27, 0.66)	−0.01 (−1.23, 1.21)	−0.04 (−0.42, 0.35)	−0.18 (−1.39, 1.02)	SOC		
0.01 (−0.51, 0.53)	0.09 (−0.42, 0.61)	0.07 (−0.57, 0.72)	0.00 (−0.52, 0.52)	−0.17 (−1.15, 0.81)	0.34 (−0.20, 0.89)	−0.14 (−1.45, 1.18)	−0.16 (−0.80, 0.48)	−0.31 (−1.61, 1.00)	−0.12 (−0.63, 0.39)	Surgical + SOC	
−0.07 (−0.21, 0.08)	0.02 (−0.11, 0.14)	0.00 (−0.42, 0.41)	−0.08 (−0.22, 0.06)	−0.25 (−1.10, 0.60)	0.27 (0.04, 0.48)	−0.21 (−1.43, 1.01)	−0.24 (−0.64, 0.16)	−0.38 (−1.60, 0.83)	−0.20 (−0.30, −0.10)	−0.08 (−0.58, 0.42)	Ultrasonic + SOC

												
Clinically important difference favoring row treatment						No difference	Clinically important difference favoring column treatment					

COO, Clostridial collagenase ointment; SOC, Standard of care; P1G10, Proteolytic Fraction from Latex of *Vasconcellea cundinamarcensis*.

### 3.4 Area reduction rate

A total of 14 RCTs evaluated the effect of different debridement procedures on the ulcer area reduction rate in patients with DFU, and a network diagram of the ulcer area reduction rate is shown in Figure 3B. The heterogeneity test indicated an I^2^ value of 9%; hence, the fixed-effects model was used. According to SUCRA analysis and the League Table (Table 3B), kiwifruit extract in enzymatic debridement had the best effect on the reduction rate of the DFU area (SUCRA = 0.931). The cumulative probability ranking graph is shown in Figure 4B, with an MD and 95% CI of 0.47 (0.27, 0.66) when compared with the SOC group.

### 3.5 Heterogeneity assessment

**Figure 5:**
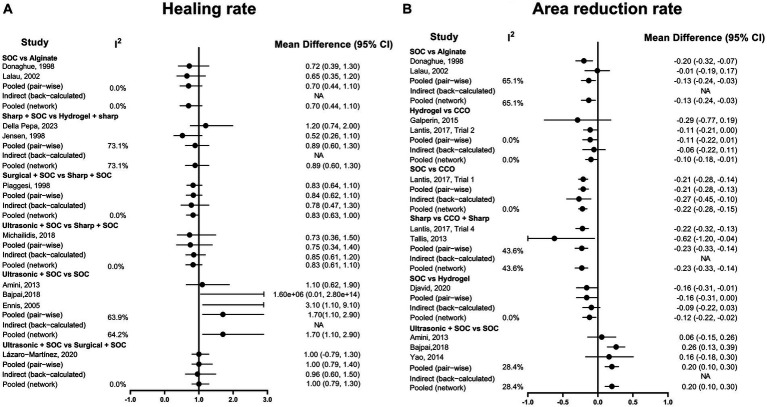
Heterogeneity assessment graph. **(A)** Healing rate; **(B)** Area reduction rate. COO, Clostridial collagenase ointment; SOC, Standard of care.

The heterogeneity test was performed separately for the two outcome indicators. For the ulcer healing rate, the heterogeneity of the network comparison of sharp + SOC vs. hydrogel + sharp was 73.1%, and the heterogeneity of the network comparison of ultrasonic + SOC vs. SOC was 64.2% (Figure 5A). Regarding the ulcer area reduction rate, the heterogeneity of the network comparison of SOC vs. alginate was 65.1% (Figure 5B). The network comparison heterogeneity of the other studies was less than 50%.

## 4 Discussion

DFU is a common complication among patients with diabetes, and its treatment options are diverse and complex. Debridement, as an essential treatment method, has garnered significant attention. To the best of our knowledge, this NMA represents the first report on an NMA comparing the healing effectiveness of various debridement methods for DFU. The debridement methods included in the final studies were mechanical debridement (sharp, surgery, or ultrasound), enzymatic debridement (clostridial collagenase ointment [COO], P1G10, or kiwifruit extract), and autolytic debridement (hydrogel or alginate). In this NMA, P1G10 debridement demonstrated the best wound healing rate among the enzymatic debridements (SUCRA = 0.919). Kiwifruit extract debridement exhibited the highest ulcer area reduction rate (SUCRA = 0.931), and COO, another enzymatic debridement, also demonstrated high effectiveness.

Enzymatic debridement, which has a long history and wide application in wound debridement for patients with burns (40–42), was found to be an excellent debridement method for DFUs in our NMA. Enzymatic debridement involves applying proteases from various sources (e.g., bacteria, plants, or animals, as seen in COO, P1G10, and kiwifruit extract) to promote the degradation of the necrotic tissue at the bottom of the wound and remove foreign bodies and secretions, thereby accelerating the wound healing process (12). Although the results of this study show that enzymatic debridement is advantageous in DFU treatment, the latest IWGDF 2023 guidelines still recommend sharp debridement as the preferred debridement method for diabetic ulcers because, although experts generally agree on the need for regular wound debridement to promote wound healing, studies presenting high-quality evidence on debridement and confirmation of the best debridement methods are still relatively limited (13). Nonetheless, the advantages of enzymatic debridement cannot be disregarded, particularly in the management of DFUs in some low-income areas that may lack skilled personnel, training programs, sterile instruments, and standard sharp debridement. Thus, in healthcare systems with such limitations, enzymatic debridement can be considered as an alternative (13). When enzyme debridement is performed for DFUs, the patient’s wound is first thoroughly evaluated, including the location, size, depth, and degree of infection of the wound. According to the nature and condition of the wound, the appropriate enzyme drugs should be selected and applied. Before enzyme debridement, the wound should be well prepared, including cleaning the wound and removing dirt. Enzymes are usually applied onto the wound surface in the form of ointments or liquids, and it is important to ensure even application onto the wound. Then, cover the wound to prevent the enzyme drug from spilling over while keeping the wound moist. The treatment process requires regular monitoring to observe changes in dead tissue and wound healing. Treatment usually needs to be performed every day or every few days, depending on the wound condition. In addition, enzyme debridement therapy is often used in combination with other treatment strategies, such as antibiotic therapy, foot pressure relief, and blood glucose management. It is essential to continuously monitor the patient’s response, including the rate of wound healing, infection, and comfort, and to adjust treatment strategies, if necessary.

In addition to sharp debridement, mechanical debridement in this NMA includes surgery and ultrasound. However, these debridement modalities have demonstrated poor effectiveness on both DFU healing and ulcer area reduction, while they are expensive; require a sterile environment, well-trained practitioners, and specific devices; and are contraindicated in patients with coagulation disorders (43). However, it cannot be denied that surgical debridement is more thorough, especially suitable for wounds with severe infections, and this approach also has the advantage of obtaining deep-tissue specimens for pathological examination. Ultrasound debridement causes less trauma than sharp debridement, thereby reducing patient discomfort. Clinicians should also be aware that, during ultrasound debridement, there is a potential risk of exposure to aerosolized microorganisms and fragments from the wound (5). In this NMA, two autolytic debridements, namely, hydrogel and alginate, were mainly included. Although autolytic debridement is a conservative treatment strategy, it did not demonstrate high effectiveness in the included studies. Chronic wounds heal slowly, depending on appropriate response conditions and the patient’s physiological response, thus increasing the risk of skin degeneration due to prolonged exposure of the surrounding skin to a moist environment (43). Unfortunately, this study did not identify any RCTs that met our inclusion criteria for wet-to-dry, hydrosurgery, and biological debridement. Wet-to-dry debridement, owing to its non-selective removal of granulation tissue, is prone to damaging healthy tissue and causing increased patient discomfort (5). Although hydrosurgery offers the advantage of shorter processing time and suitability for larger wounds, it may pose a risk of cross-contamination, and research on hydrosurgery is very limited (8). Regarding biological debridement, one RCT on maggot debridement was published as a meeting abstract without peer review (44). In 2019, an RCT (45) reported on maggot debridement, but the study mainly aimed to determine the outcome of inflammatory indicators, which did not align with the outcome of the present study; hence, it was also excluded. However, biological debridement, which involves the digestive action of sterile maggots from *Lucilia sericata* to remove devitalized epithelial cells (46), has shown effectiveness in some chronic ulcers (47). Nevertheless, societal negative perceptions of maggots have hindered the acceptance of this option among patients and practitioners. At the same time, it is essential to recognize that different debridement methods may yield different results in various patients and different conditions. Therefore, clinicians should assess the advantages and complementarity of different debridement approaches and apply them reasonably in clinical practice. When formulating a treatment plan, clinicians should perform a comprehensive assessment based on the patient’s specific conditions, ulcer characteristics, and feasibility to ensure the most suitable treatment for each individual.

It is essential to acknowledge that all the studies included in this NMA had important methodological limitations, primarily stemming from the lack of blinding in most studies, resulting in a high risk of bias that substantially reduces the reliability of the results. When assessing heterogeneity, our NMA revealed notable mesh comparison heterogeneity in certain cases. For instance, the comparison of SOC + sharp vs. hydrogel + sharp in terms of ulcer healing rate exhibited 73.1% heterogeneity. In this context, Della Pepa’s study relied on patients’ daily home dressing changes without blinded treatment, while Jensen’s study was relatively old, not strictly randomized, and carried a high risk of bias. Similarly, the heterogeneity of ultrasonic + SOC vs. SOC in terms of ulcer healing rate was 64.2%. Both Bajpai and Ennis studies showed significantly higher healing rates in the ultrasonic + SOC group, with both studies having the same follow-up duration (3 months) and relatively high quality. By contrast, Amini’s study found similar healing rates between ultrasonic + SOC and SOC groups, which may be attributed to the relatively long follow-up time (6 months) and the fact that the ulcers were mostly healed. Furthermore, Amini’s study lacked strict randomization and blinding, and its quality was low. In the network comparison of ulcer area reduction rate, the heterogeneity of alginate vs. SOC was 65.1%. Studies conducted by Donaghue and Lalau were relatively outdated and had a high risk of bias. Donaghue’s study might have had selective reporting, leading to publication bias, while Lalau’s study had a significantly shorter follow-up duration than Donaghue’s study. Therefore, to validate these findings, future research should focus on conducting more rigorous, high-quality, large-sample RCTs.

This NMA has several limitations. The varied length of the study, follow-up time, debridement frequency, and definitions of healing affected the outcome indicators of healing rate and ulcer area reduction rate. Additionally, more than half of the included studies had a high risk of bias, suggesting caution when applying the results to clinical practice. Moreover, because of the limitations in data extraction from the included literature, this study discussed only the comparison of different debridement methods for DFUs in terms of the ulcer healing rate and ulcer area reduction rate and did not address the safety and cost-effectiveness of these methods.

## 5 Conclusion

Although sharp debridement remains the preferred debridement method for most DFUs globally, there is mounting evidence supporting the advantages of enzymatic debridement in ulcer healing rate and ulcer area reduction rate. However, given the current low research quality, enzymatic debridement should be considered as a candidate debridement method alongside sharp debridement in clinical practice. To confirm these results, future research should focus on more rigorous, high-quality, large-sample RCTs. Clinicians should conduct a comprehensive assessment based on each patient’s specific conditions, ulcer characteristics, and feasibility to provide the most appropriate treatment.

## Data availability statement

The original contributions presented in the study are included in the article/Supplementary material, further inquiries can be directed to the corresponding authors.

## Author contributions

PN: Data curation, Funding acquisition, Methodology, Writing – original draft. YL: Writing – review & editing. JK: Writing – review & editing. HC: Funding acquisition, Resources, Supervision, Writing – review & editing. JZ: Methodology, Writing – review & editing.
