# Supplementary material for: Comparison of healing effectiveness of different debridement approaches for diabetic foot ulcers: a network meta-analysis of randomized controlled trials
Source: Front Public Health. 2023 Dec 11;11:1271706. doi: 10.3389/fpubh.2023.1271706 (PMC10749485; doi:10.3389/fpubh.2023.1271706)
Supplement: Supplementary file 1 [file Table_1.DOCX]

| **PubMed(n=563)** | **Embase(n=991)** | **Cochrane Library(n=927)** |
| --- | --- | --- |
| #1 diabetic foot[MeSH Terms] | #1 'diabetic foot'/exp | #1 MeSH descriptor: [Diabetic Foot] explode all trees |
| #2 "diabetic feet" OR "diabetic foot" OR "diabetic foot syndrome" OR "diabetic foot ulcer" | #2 'diabetic feet' OR 'diabetic foot' OR 'diabetic foot syndrome' OR 'diabetic foot ulcer' | #2 'diabetic feet' OR 'diabetic foot' OR 'diabetic foot syndrome' OR 'diabetic foot ulcer' |
| #3 debridement*[MeSH Terms] | #3 'debridement'/exp | #3 MeSH descriptor: [Debridement] explode all trees |
| #4 "cleanse" OR "mechanical" OR "surgery" OR "surgical" OR "scalpel" OR "sharp" OR "ultrasound" OR "laser*" OR "irrigate" OR "irrigation" OR "whirlpool" OR "autolytic " OR "autolyses" OR "hydrogel" OR "in situ hydrogel*" OR "patterned hydrogel*" OR "gel*" OR "alginate" OR "kaltostat" OR "vocoloid" OR "calginat" OR "potassium alginate" OR "sodium alginate" OR "kalrostat 2" OR "sodium calcium alginate" OR "barium alginate" OR "calcium alginate" OR "copper alginate" OR "alloid G" OR "kalrostat" OR "xantalgin" OR "enzymatic" OR "bromelin*" OR "bromelain" OR "dayto anase" OR "debrase" OR "dontisanin" OR "ananase" OR "mucozym" OR "proteozym" OR "traumanase" OR "bromelain POS" OR "extranase" OR "papain" OR "tromasin" OR "collagen degrading enzyme" OR "collagenase" OR "collagen peptidase" OR "trypsin" OR "tripcellim" OR "trypure" OR "beta trypsin" OR "subtilis proteinase" OR "human fibrinolytic enzyme" OR "chymotrypsin" OR "alpha chymotrypsin choay" OR "avazyme" OR "biosurgery" OR "larvae" OR "maggot*" OR "tadpole*" | #4 'cleanse' OR 'mechanical' OR 'surgery' OR 'surgical' OR 'scalpel' OR 'sharp' OR 'ultrasound' OR 'laser*' OR 'irrigate' OR 'irrigation' OR 'whirlpool' OR 'autolytic' OR 'autolyses' OR 'hydrogel' OR 'in situ hydrogel*' OR 'patterned hydrogel*' OR 'gel*' OR 'alginate' OR 'kaltostat' OR 'vocoloid' OR 'calginat' OR 'potassium alginate' OR 'sodium alginate' OR 'kalrostat 2' OR 'sodium calcium alginate' OR 'barium alginate' OR 'calcium alginate' OR 'copper alginate' OR 'alloid g' OR 'kalrostat' OR 'xantalgin' OR 'enzymatic' OR 'bromelin*' OR 'bromelain' OR 'dayto anase' OR 'debrase' OR 'dontisanin' OR 'ananase' OR 'mucozym' OR 'proteozym' OR 'traumanase' OR 'bromelain pos' OR 'extranase' OR 'papain' OR 'tromasin' OR 'collagen degrading enzyme' OR 'collagenase' OR 'collagen peptidase' OR 'trypsin' OR 'tripcellim' OR 'trypure' OR 'beta trypsin' OR 'subtilis proteinase' OR 'human fibrinolytic enzyme' OR 'chymotrypsin' OR 'alpha chymotrypsin choay' OR 'avazyme' OR 'biosurgery' OR 'larvae' OR 'maggot*' OR 'tadpole*' | #4 'cleanse' OR 'mechanical' OR 'surgery' OR 'surgical' OR 'scalpel' OR 'sharp' OR 'ultrasound' OR 'laser*' OR 'irrigate' OR 'irrigation' OR 'whirlpool' OR 'autolytic ' OR 'autolyses' OR 'hydrogel' OR 'in situ hydrogel*' OR 'patterned hydrogel*' OR 'gel*' OR 'alginate' OR 'kaltostat' OR 'vocoloid' OR 'calginat' OR 'potassium alginate' OR 'sodium alginate' OR 'kalrostat 2' OR 'sodium calcium alginate' OR 'barium alginate' OR 'calcium alginate' OR 'copper alginate' OR 'alloid G' OR 'kalrostat' OR 'xantalgin' OR 'enzymatic' OR 'bromelin*' OR 'bromelain' OR 'dayto anase' OR 'debrase' OR 'dontisanin' OR 'ananase' OR 'mucozym' OR 'proteozym' OR 'traumanase' OR 'bromelain POS' OR 'extranase' OR 'papain' OR 'tromasin' OR 'collagen degrading enzyme' OR 'collagenase' OR 'collagen peptidase' OR 'trypsin' OR 'tripcellim' OR 'trypure' OR 'beta trypsin' OR 'subtilis proteinase' OR 'human fibrinolytic enzyme' OR 'chymotrypsin' OR 'alpha chymotrypsin choay' OR 'avazyme' OR 'biosurgery' OR 'larvae' OR 'maggot*' OR 'tadpole*' |
| #5 randomized controlled trial[MeSH Terms] | #5 'randomized controlled trial'/exp | #5 MeSH descriptor: [Randomized Controlled Trial] explode all trees |
| #6 "randomised controlled study" OR "randomised controlled trial" OR "randomized controlled study" OR "randomized controlled trial" | #6 'randomised controlled study' OR 'randomised controlled trial' OR 'randomized controlled study' OR 'randomized controlled trial' | #6 'randomised controlled study' OR 'randomised controlled trial' OR 'randomized controlled study' OR 'randomized controlled trial' |
| #7 (#1 OR #2) AND (#3 OR #4) AND (#5 OR #6) | #7 (#1 OR #2) AND (#3 OR #4) AND (#5 OR #6) | #7 (#1 OR #2) AND (#3 OR #4) AND (#5 OR #6) |

**Table S1** Complete list of 3 electronic library search terms.
